# Adverse prognosis of glioblastoma contacting the subventricular zone: Biological correlates

**DOI:** 10.1371/journal.pone.0222717

**Published:** 2019-10-11

**Authors:** Sharon Berendsen, Emma van Bodegraven, Tatjana Seute, Wim G. M. Spliet, Marjolein Geurts, Jeroen Hendrikse, Laurent Schoysman, Willemijn B. Huiszoon, Meri Varkila, Soufyan Rouss, Erica H. Bell, Jérôme Kroonen, Arnab Chakravarti, Vincent Bours, Tom J. Snijders, Pierre A. Robe

**Affiliations:** 1 UMC Utrecht Brain Center, Department of Neurology and Neurosurgery, University Medical Center of Utrecht, Utrecht, The Netherlands; 2 UMC Utrecht Brain Center, Department of Translational Neuroscience, University Medical Center of Utrecht, Utrecht, The Netherlands; 3 Department of Pathology, University Medical Center of Utrecht, Utrecht, The Netherlands; 4 Department of Radiology, University Medical Center of Utrecht, Utrecht, The Netherlands; 5 Department of Human Genetics, GIGA Research Center, Liège University Hospital, Liège, Belgium; 6 Department of Radiology, Liège University Hospital, Liège, Belgium; 7 Department of Radiation Oncology, Wexner Medical Center, James Cancer Center, Ohio State University, Columbus, OH, United States of America; University of Pécs Medical School, HUNGARY

## Abstract

**Introduction:**

The subventricular zone (SVZ) in the brain is associated with gliomagenesis and resistance to treatment in glioblastoma. In this study, we investigate the prognostic role and biological characteristics of subventricular zone (SVZ) involvement in glioblastoma.

**Methods:**

We analyzed T1-weighted, gadolinium-enhanced MR images of a retrospective cohort of 647 primary glioblastoma patients diagnosed between 2005–2013, and performed a multivariable Cox regression analysis to adjust the prognostic effect of SVZ involvement for clinical patient- and tumor-related factors. Protein expression patterns of a.o. markers of neural stem cellness (CD133 and GFAP-δ) and (epithelial-) mesenchymal transition (NF-κB, C/EBP-β and STAT3) were determined with immunohistochemistry on tissue microarrays containing 220 of the tumors. Molecular classification and mRNA expression-based gene set enrichment analyses, miRNA expression and SNP copy number analyses were performed on fresh frozen tissue obtained from 76 tumors. Confirmatory analyses were performed on glioblastoma TCGA/TCIA data.

**Results:**

Involvement of the SVZ was a significant adverse prognostic factor in glioblastoma, independent of age, KPS, surgery type and postoperative treatment. Tumor volume and postoperative complications did not explain this prognostic effect. SVZ contact was associated with increased nuclear expression of the (epithelial-) mesenchymal transition markers C/EBP-β and phospho-STAT3. SVZ contact was not associated with molecular subtype, distinct gene expression patterns, or markers of stem cellness. Our main findings were confirmed in a cohort of 229 TCGA/TCIA glioblastomas.

**Conclusion:**

In conclusion, involvement of the SVZ is an independent prognostic factor in glioblastoma, and associates with increased expression of key markers of (epithelial-) mesenchymal transformation, but does not correlate with stem cellness, molecular subtype, or specific (mi)RNA expression patterns.

## Introduction

Glioblastoma is the most malignant primary brain tumor, with a median prognosis of 15–20 months despite intensive treatment [1]. In many patients, glioblastoma cells invade the subventricular zone (SVZ) [2, 3]. This area represents a neurogenic zone in the adult brain and contains neural stem cells [4], which are suggested to play a role in gliomagenesis [4–6]. It is also a protective niche attracting tumor-initiating cells and allowing them to escape treatment [4, 7–11] and could thus favor tumor progression [12–14]. Furthermore, a more invasive and multifocal phenotype of tumors contacting the SVZ on MRI was reported [15].

Based on univariable statistics [14, 16, 17] or small to mid-size patient series [17, 18], the radiological involvement of the SVZ seems to associate with an adverse prognosis. Radiogenomics [19–23] and proteomics [24] studies have proposed potential associations between MRI characteristics and gene/protein expression profiles in glioblastoma. These studies have variably associated SVZ-contacting tumors with differential expression of several genes and gene expression signatures, involving glioma stem cell signaling, hypoxia, tumor vascularity, and invasion [19–26]. Simple radiological features might thus be informative of the tumor’s biological characteristics.

In this paper, we aim to validate the prognostic role of glioblastoma involvement of the SVZ in a large, well-characterized cohort of 647 patients. Additionally, we analyze clinical and tumor biological factors that associate with this prognostic effect, to help further understand this relation.

## Materials and methods

### Ethics statement

This study was conducted following approval by the local ethical committee (METC Utrecht) and institutional review board (Biobank Research Ethics Committee Utrecht, protocols 16–229, 16–342, 16–348). Fresh-frozen samples were obtained following written informed consent. According to Dutch regulations, the need for informed consent was waived for the rest of this retrospective analysis.

### Patient cohorts

**Fig 1:**
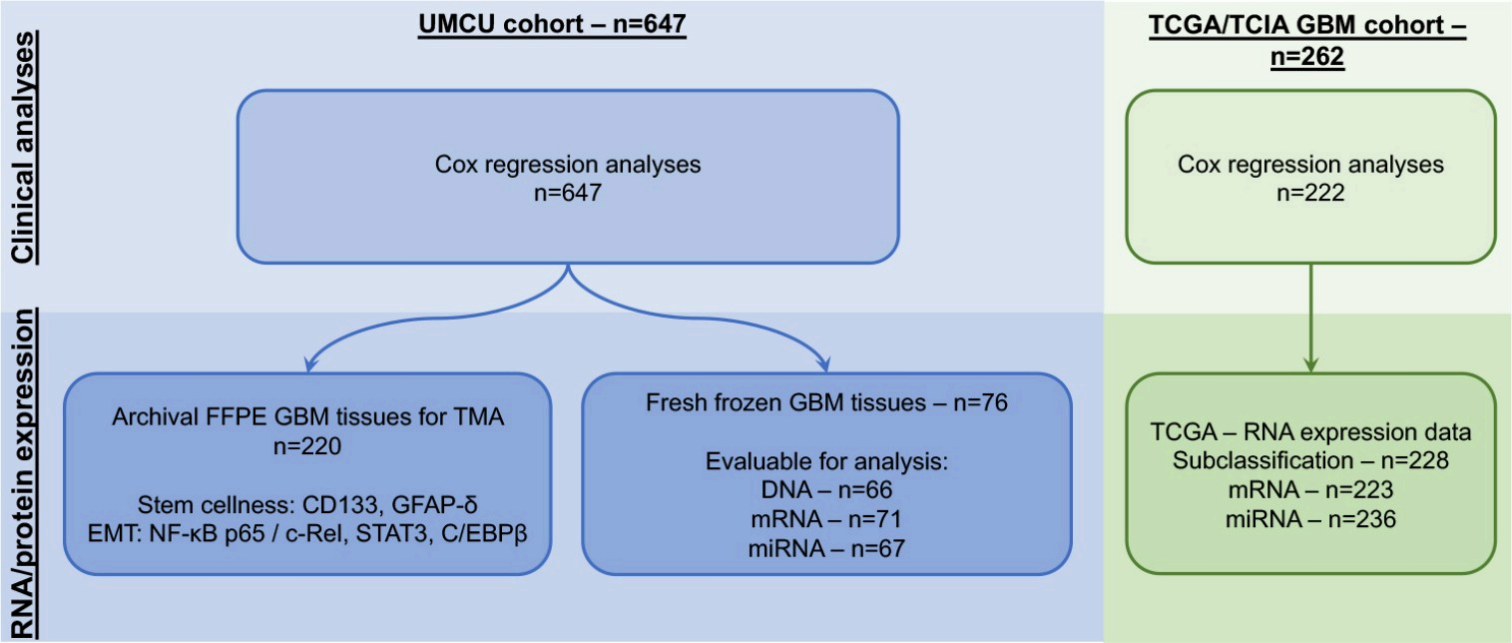
Overview of study cohorts. Abbreviations: UMCU: University Medical Center Utrecht; FFPE: Formalin fixed paraffin embedded; GBM: glioblastoma; EMT: epithelial-mesenchymal transition; TCGA: The Cancer Genome Atlas; TCIA: The Cancer Imaging Archive.

A flowchart describing the cohorts in this study is included in Fig 1.

All adult patients (n = 647) with histologically confirmed *de novo* supratentorial glioblastoma (WHO grade IV) diagnosed at the University Medical Center of Utrecht (UMCU) between 2005–2013 were retrospectively included. Details on this cohort are provided in Table 1, S1 Appendix and were published before [27]. Age, gender, Karnofsky performance status (KPS), tumor volume, surgery type and postoperative treatment were recorded. Complications within 30 days from surgery were recorded according to the Common Terminology Criteria for Adverse Events (CTCAE). Survival data (days from surgery) were retrieved from hospital records and community archives. IDH1 mutational status was not yet routinely determined in clinical practice at our center, and was available for 343/647 patients from a previous study [27]. SVZ contact was defined as direct contact of the T1-weighted, gadolinium-enhancing part of the tumor with the lateral ventricles, and as detailed in the supplementary methods (S2 Appendix). Tumor volume was measured as the volume of the contrast-enhancing lesion on the presurgical MRI scan with OsiriX software version 4.1.2 (Pixmeo, Bernex, Switzerland).

**Table 1:** Baseline table.

Patient characteristics	SVZ contact	No SVZ contact	Statistical analysis
*n (%)*	371 (57.3)	240 (37.1)	
**Age *(mean ± SD)***	62.0 ± 12.1	60.6 ± 12.7	Independent t-test, *P* = 0.16
**Gender *(% male)***	60.6	60.0	χ^2^ test, *N*.*S*.
**KPS *n (%)* < 70**	112 (30.2)	50 (20.8)	χ^2^ test, *P* = 0.01
**≥ 70**	256 (69.0)	189 (78.8)	
	Missing: 3 (0.8)	Missing: 1 (0.4)	
**Tumor volume *cm*^*3*^**	46.8 (27.8–73.1)	16.8 (6.9–32.8)	Mann Whitney U test,
***(median (IQR))***	*Missing*: *6 (1*.*6)*	*Missing*: *3 (1*.*3)*	*P* < 0.0005
**Extent of surgery *n (%)***			Fisher exact test,
**Biopsy**	154 (41.5)	55 (22.9)	*P* < 0.0005
**Debulking**	217 (58.5)	185 (77.1)	
**Post-surgical treatment *n (%)***			Fisher exact test,
**None**	97 (26.1)	34 (14.2)	*P* < 0.0005
**Monotherapy**	101 (27.2)	52 (21.7)	
**RT + TMZ**	171 (46.1)	152 (63.3)	
	*Missing*: *2 (0*.*5)*	*Missing*: *2 (0*.*8)*	
**CTCAE complications** **grade 3–5 *n (%)***	62 (16.7)	25 (10.4)	χ^2^ test, *P* = 0.032
**IDH1 mutational status *n (%)***			χ^2^ test, *P* = 0.21
**R132H mutation**	9 (2.4)	12 (5.0)	
**Wild-type**	183 (49.3)	139 (57.9)	
	*Missing*: *179 (48*.*2)*	*Missing*: *89 (37*.*1)*	

Missing SVZ status: 36 (5.6%). Abbreviations: KPS: Karnofsky performance score; SVZ: subventricular zone; IQR: interquartile range; RT: radiotherapy; TMZ: temozolomide

From these patients, 76 fresh-frozen surgical samples of *de novo* glioblastomas were prospectively collected between 2010 and 2015 for DNA, mRNA and miRNA analyses. SVZ status was unavailable for 5 of these patients.

In addition, we retrospectively collected archival tumor tissues for the consecutive 229 glioblastoma patients treated in the UMCU between 2005 and 2008. Tissue was available for inclusion in tissue microarrays (TMAs) for 220/229 patients. SVZ status was unavailable for 14 of these patients.

### mRNA and miRNA expression analysis

Processing of the fresh-frozen surgical samples-derived mRNA and miRNA samples and data was described previously [28]. Analyses are described in more detail in the supplementary methods (S2 Appendix). Microarray data are made publically available on the GEO platform (accession number GSE134783).

After omitting samples with missing MRI data or insufficient RNA quality, 71 RNA samples and 67 miRNA samples were evaluable for analysis. Gene expression analyses were perfomed using the Partek suite built-in ANOVA pipeline, with an FDR<0.05 treshold for significancy. Exploratory Gene Set Enrichment Analyses (GSEA) were performed with use of the Broad Institute MySig libraries of curated gene sets C1 –C7 version 5.0 [29]. An exploratory false discovery rate (FDR) threshold of 0.25 was applied as recommended [30]. Molecular subclassification (proneural, neural, classical, mesenchymal) was predicted by hierarchical clustering, as described previously [28, 31]. Classes could be unequivocally assigned to 62 samples.

For the miRNA analyses, RNA was isolated with the MiRNeasy Micro Kit (Qiagen, Venlo, The Netherlands). Expression profiling of 800 miRNA probes was performed with the nCounter Human v2 miRNA Expression Assay (NanoString Technologies, Seattle, USA) at The Ohio State University Nucleic Acid Core Facility.

### Copy number analysis

DNA was extracted with sufficient quality from 67 fresh-frozen samples of our proprietary cohort of glioblastomas and processed on SNP6.0 Affymetrix chips according to manufacturer’s recommendations. One outlier was removed after principal component analysis, and 66 samples were evaluable for analysis. Further details are described in the supplementary methods (S2 Appendix).

### Tissue microarrays and immunohistochemistry

TMAs were constructed including archival glioblastoma tissue from 220 patients, and were processed as reported previously [27]. Immunohistochemistry was performed, as described previously [27, 28] and in the supplementary methods (S2 Appendix), with antibodies against c-Rel, NF-κB p65 (phospho-S276), STAT3 (phospho-Y705), anti-C/EBPβ, anti-CD133 and anti-GFAP-δ.

Protein expression evaluation was evaluated with blinding to the clinical data, under supervision of a senior neuropathologist (WS). The percentage of nuclear and/or cytoplasmatic staining was scored on triplicate tumor cores as: 0, negative; 1, 1–25% positive cells; 2, 26–50% positive cells; 3, 51–75% positive cells and 4, 76–100% positive cells.

The percentage of positive GFAP-δ staining was calculated per sample. A mean staining score was computed per patient. Scores were analyzed with Mann Whitney U tests.

### Analysis of TCGA/TCIA MRI and mRNA / miRNA expression data

Preoperative gadolinium-enhanced T1-weighted MRI scans from the The Cancer Genome Atlas (TCGA) glioblastoma patients were downloaded from The Cancer Imaging Archive (TCIA; September 2015) and assessed for SVZ contact as described above. Researchers were blinded to the clinical data. A total of 222 patients could be included in the Cox regression analyses. Corresponding TCGA molecular classification and gene expression data were obtained for 228 and 223 patients, as described above and in the supplementary methods (S2 Appendix). Level 3 miRNA expression data from 236 glioblastomas was downloaded from the TCGA data portal (December 2015). MiRNA expression levels in glioblastomas with and without SVZ contact were analyzed with the ‘limma’ and ‘heatmap3’ package (R v3.2.2).

### Survival analyses

Statistical analyses were performed with use of SPSS 25.0 (IBM, Armonk, USA). All statistical tests were two-sided, and a P value < 0.05 was considered statistically significant.

Kaplan-Meier curves were analyzed with the log-rank test. Cox regression was used for the survival analyses. The proportional hazards assumption of the Cox model was tested (details in supplementary methods (S2 Appendix)) [27]. Multivariable Cox regression was performed including the variables SVZ status, age, KPS, type of surgery, adjuvant treatment and a time-dependent variable for KPS. In a multivariable complete case analysis, 595 patients could be included. As a sensitivity analysis, multiple imputation was performed to include all 647 patients. Next, a Cox regression analysis was performed including the variables tumor volume, type of surgery, postoperative treatment, CTCAE grade 3–5 complications and time-dependent variables for KPS and tumor volume to explore underlying factors that could influence the observed prognostic effect. Missing values were imputed in this analysis.

Survival analyses with TCGA/TCIA data were performed as described above. Multivariable Cox regression was performed including age and KPS variables in the model. Multiple imputation based on these variables was performed for missing data, to include all 222 TCGA/TCIA-glioblastoma patients of which survival and MRI data was available.

## Results

### SVZ contact is an independent prognostic factor in glioblastoma

**Fig 2:**
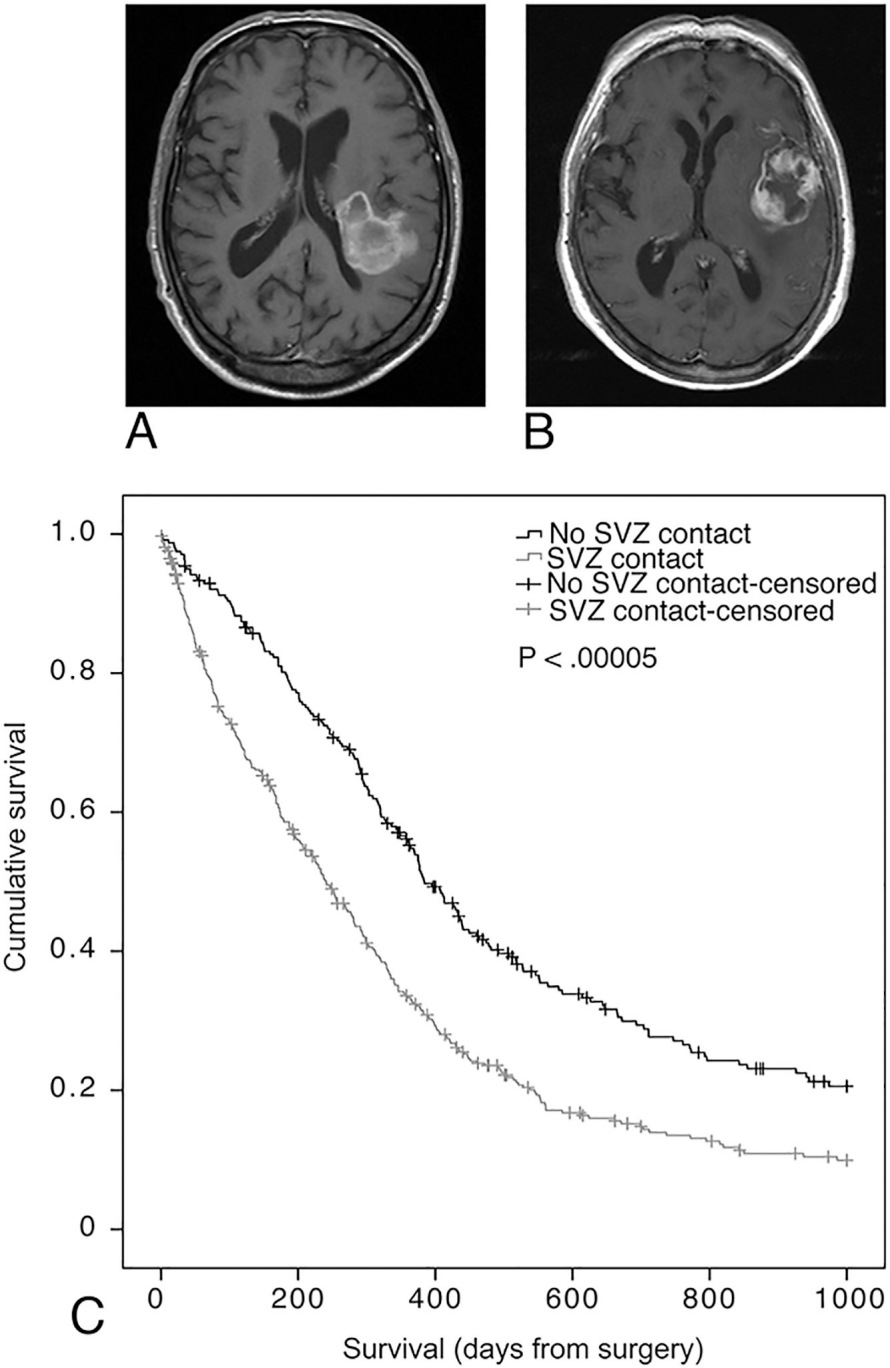
SVZ involvement associates with glioblastoma patient survival. **A.** Pre-operative T1-weighted MRI scan with gadolinium of patient (M, 1928) with glioblastoma contacting the SVZ. **B.** Pre-operative T1-weighted MRI scan with gadolinium of patient (F, 1925) with glioblastoma not contacting the SVZ. **C.** Association of tumor contact with the SVZ on glioblastoma patient survival. Kaplan-Meier plot of glioblastoma patients with a tumor contacting the SVZ (grey) and patients with a tumor that does not contact the SVZ (black). Patients with survival over 1000 days from surgery were censored. Survival was significantly different between the two groups (log-rank test, *P* < 0.00005).

SVZ status could not be determined for 36 patients (5.6%) of our cohort, due to unavailable MRI scans. Of the remaining 611 glioblastoma patients, 371 (57.3%) had an SVZ-contacting tumor on the preoperative MRI (Fig 2A and 2B). SVZ-contacting tumors were significantly associated with worse prognosis (median survival: 241 days from surgery; 95%CI: 203.6 to 278.4) compared to tumors without SVZ contact (median survival: 384 days; 95%CI: 338.9 to 429.1, log-rank test (Fig 2C; P<0.0005), unadjusted HR:1.70; 95%CI:1.40 to 2.05, P<0.0005, Table 2). Multivariable complete case Cox regression (n = 595) with correction for age, preoperative KPS, type of surgery and adjuvant treatment showed that SVZ contact remained independently associated with worse overall survival (Adjusted HR: 1.57; 95%CI: 1.29 to 1.91; P<0.0005; Table 2). Multiple imputation allowed for inclusion of all patients (n = 647) in this multivariable analysis and did not alter the results (Adjusted HR:1.50; 95%CI: 1.24 to 1.82; P<0.0005). IDH1 mutational status was only available for a subset of the total cohort (n = 343 of 647 patients) and did not correlate with SVZ contact (Chi-square test, p = 0.21, Table 1).

**Table 2:** Cox regression analysis–prognostic model.

**Variable**	**Crude HR** **(95% CI)**	***P*-value**	**Adjusted HR** **(95% CI)**	***P*-value**
**SVZ contact**	1.70 (1.40–2.05)	< 0.0005	1.57 (1.29–1.91)	< 0.0005
**Gender (male)**	1.05 (0.88–1.26)	0.57	-	-
**Age**	1.03 (1.03–1.04)	< 0.0005	1.01 (1.01–1.02)	0.002
**KPS < 70**	1.00		1.00	
**KPS ≥ 70**	0.44 (0.36–0.53)	< 0.0005	0.53 (0.37–0.74)	< 0.0005
KPS * time			1.002 (1.001–1.003)	0.003
**Biopsy**	1.00		1.00	
**Resection**	0.39 (0.33–0.47)	<0.0005	0.65 (0.53–0.80)	<0.0005
**Adjuvant treatment**				
None	1.00		1.00	
RT only	0.15 (0.11–0.19)	<0.0005	0.20 (0.15–0.26)	<0.0005
RT + TMZ	0.05 (0.04–0.06)	<0.0005	0.08 (0.06–0.11)	<0.0005

Abbreviations: HR: hazard ratio; KPS: Karnofsky performance score; SVZ: subventricular zone; RT: radiotherapy; TMZ: temozolomide.

SVZ contact was also a negative prognostic factor in the TCGA/TCIA dataset both in univariable analyses (log-rank *P*<0.05; Unadjusted HR: 1.43; 95%CI: 1.06 to 1.91; *P*<0.05) and after correction for age and KPS (Adjusted HR:1.37; 95%CI: 1.02 to 1.84; *P*<0.05).

### Patient- and tumor-related clinical factors do not explain the prognostic effect of SVZ contact in glioblastoma

SVZ-contacting tumors exhibited larger tumor volumes compared to tumors without SVZ contact (Mann-Whitney U test, P<0.0005, Table 1). Patients with a glioblastoma contacting the SVZ had a lower preoperative KPS (χ^2^-test; P = 0.01), underwent more biopsy procedures (P<0.0005) and were less often treated with adjuvant chemoradiation (P<0.0005). In addition, 16.7% of patients with an SVZ-contacting tumor experienced at least one CTCAE grade 3–5 complication in the 30-day postoperative period, compared to 10.5% of patients with a tumor without SVZ contact (χ^2^-test; P<0.05, Table 1). No statistically significant difference in the prevalence of postoperative cerebral hemorrhage/ischemia, epilepsy, infection/meningitis, hydrocephalus or thrombosis. Patients with an SVZ-contacting tumor did more often experience metabolic complications, such as hyponatremia (P = 0.005), and hyperglycemia (P<0.05).

After inclusion of these factors in the multivariable Cox model, SVZ contact remained a significant prognostic factor in glioblastoma patients (adjusted HR: 1.42; 95%CI: 1.13 to 1.77, P<0.01, Table 3).

**Table 3:** Cox regression analyses–explanatory multivariable analysis.

Variable	Crude HR (95% CI)	*P*-value	Adjusted HR (95% CI)	*P*-value
**SVZ contact**	1.70 (1.40–2.05)	< 0.0005	1.42 (1.13–1.77)	0.003
**Age**	1.03 (1.03–1.04)	< 0.0005	1.02 (1.01–1.02)	< 0.0005
**KPS < 70**	1.00		1.00	
**KPS ≥ 70**	0.44 (0.36–0.53)	< 0.0005	0.56 (0.40–0.78)	0.001
KPS * time			1.002 (1.001–1.003)	0.002
**Biopsy**	1.00		1.00	
**Resection**	0.39 (0.33–0.47)	< 0.0005	0.58 (0.47–0.72)	< 0.0005
**Adjuvant treatment**				
None	1.00		1.00	
RT only	0.15 (0.11–0.19)	< 0.0005	0.18 (0.14–0.25)	< 0.0005
RT + TMZ	0.05 (0.04–0.06)	< 0.0005	0.08 (0.06–0.12)	< 0.0005
**Tumor volume**	1.002 (0.999–1.005)	0.13	1.009 (1.005–1.014)	< 0.0005
Tumor volume * time			1.000 (1.000–1.000)	< 0.0005
**Complications grade CTCAE 3–5**	0.55 (0.44–0.70)	< 0.0005	1.21 (0.94–1.55)	0.14

Abbreviations: HR: hazard ratio; KPS: Karnofsky performance score; SVZ: subventricular zone; RT: radiotherapy; TMZ: temozolomide.

### SVZ contact, gene, and miRNA expression pattern

We analyzed the mRNA expression in 71 UMCU glioblastoma samples (39 (54.9%) with SVZ contact and 32 (45.1%) without SVZ contact) and 223 TCGA/TCIA samples (128 (57%) with SVZ contact and 96 (43%) without SVZ contact). The molecular subtype distribution of the glioblastomas [31] was not different between the groups in both the UMCU (Fisher’s exact test, P = 1.0) and TCGA/TCIA datasets (χ^2^-test, P = 0.11, S1 Fig). After correction for multiple testing (FDR<0.05), no differentially expressed single gene was detected between the groups in both datasets (S2 and S3 Figs). Likewise, no miRNA was differentially expressed between the two groups in both UMCU and TCGA/TCIA cohorts (S4 and S5 Figs).

Exploratory GSEA with the C1 positional gene sets showed an enrichment of the chr9q34 gene set in tumors without SVZ contact (P<0.001, FDR = 0.038) in the UMCU dataset and downregulation of gene sets corresponding to cytogenetic bands chr3q22–23, chr3p24-25, chr3q26-29 and chr19p12 (*P*<0.01; FDR<0.25) in tumors without SVZ contact in the TCGA/TCIA data (S1 Table). The DNA analysis of our samples did however not reveal any significant difference in copy number in any area of the genome between both groups (FDR>0.5 for all cytobands).

Tumors without SVZ contact also presented an increased activation of genes with promoter regions containing the motifs GTCNYYATGR (unknown target, P<0.001, FDR = 0.24) and GCGNNANTTCC (P = 0.002, FDR = 0.23), which is an NF-κB C-rel regulatory motif [32] in the UMCU cohort. Differential activation of genes containing these regulatory motifs was not confirmed in TCGA/TCIA tumors, which only showed an enrichment of genes upregulated by Bmi-1 knockdown in tumors without SVZ contact (P = 0.004, FDR = 0.19). Bmi-1 is known to induce NF-κB signaling in glioma cells [33].

### Differential activation of proteins involved in (epithelial-) mesenchymal transformation in glioblastomas contacting the SVZ

**Fig 3:**
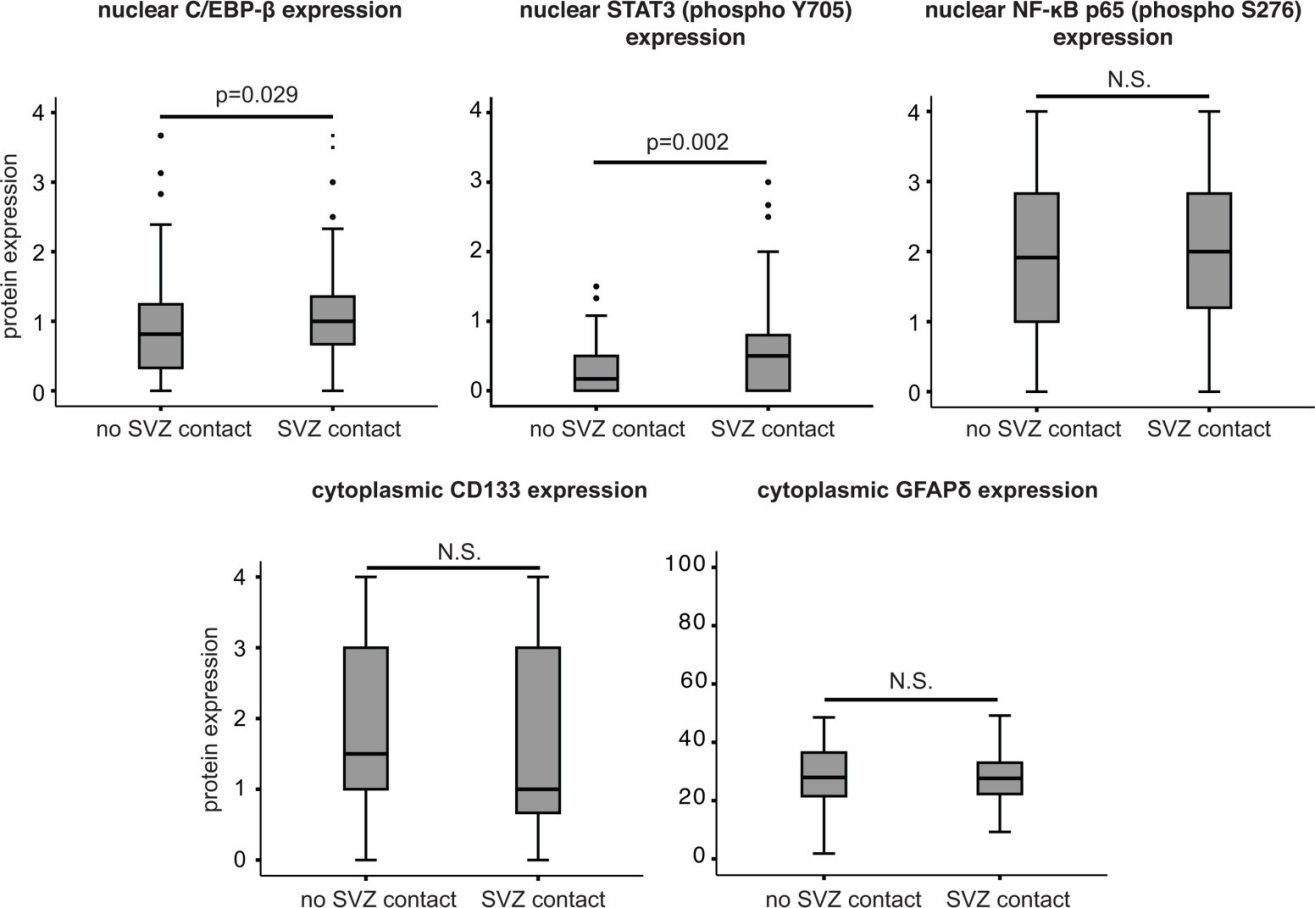
SVZ involvement in glioblastoma correlates with increased protein expression of key markers of (epithelial-)mesenchymal transformation. Increased protein expression levels of (epithelial-)mesenchymal transformation markers C/EBP-β (Mann Whitney U test, *P* = 0.029) and P^Y705^-STAT3 (Mann Whitney U test, *P* = 0.002), but not P^S536^-p65, were observed in glioblastomas contacting the SVZ. No expression differences in stem cell markers CD133 or GFAP-δ were observed between tumors with or without SVZ contact.

Since neural stem cells are a core component of the SVZ, it can be hypothesized that their presence might explain the adverse prognostic effect of SVZ contact in glioblastomas. Also, activation of mesenchymal genes is associated with poor prognosis in glioblastoma patients [34]. This may not always be fully recapitulated by gene expression data. Therefore, we analyzed the protein expression patterns of neural stem cell markers CD133 [35] and GFAP-δ [36], and the activation of key markers of (epithelial-) mesenchymal transformation C/EBP-β, NF-κB (P^S536^-p65 and c-Rel) and STAT3 [34] (Fig 3) on FFPE tumor tissue obtained from a cohort of consecutive glioblastoma patients. Baseline characteristics of this cohort were comparable to the overall dataset. No differences in CD133 (n = 141 evaluable tumors) or GFAP-δ (n = 147) expression were observed between tumors with or without SVZ contact. In contrast, we observed increased nuclear expression levels of C/EBP-β (n = 191, Mann Whitney U test, *P* = 0.029) and P^Y705^-STAT3 (n = 192, Mann Whitney U test, P = 0.002), but not of NF-κB subunits P^S536^-p65, or c-Rel in SVZ-contacting tumors.

## Discussion

In a large institutional cohort (n = 647) and a cohort from the TCIA/TCGA repository (n = 222), we show that contact between the gadolinium-enhancing, T1-weighted imaging core component of glioblastomas and the SVZ of the brain is an adverse prognostic factor, independent of other known prognostic factors age, preoperative KPS, surgery type and postoperative treatment [37]. These results considerably strengthen the associations found by others, who had explored this hypothesis with univariable survival models [14, 16, 17, 38–40] or smaller to mid-size cohorts [17, 18, 41–46], and may help define the prognosis of individual patients based on their preoperative MR imaging. A meta-analysis of these published series also showed a significant adverse prognostic effect of SVZ contact in glioblastoma patients [47], but these analyses were not corrected for the effects of other (clinical) factors, due to unavailable individual patient data. Our findings in multivariable analyses in two large cohorts, extend and validate these previous results [18, 43, 47], and point towards a true independent prognostic effect. Most importantly, we observed that this prognostic effect was independent from tumor volume and postoperative complications. In a recent study of 35 glioblastoma patients is was shown that O-(2-[18F]fluoroethyl)-L-tyrosine (FET) PET scans can show SVZ infiltration of glioblastomas that is not visible on MRI scans, which is correlated to larger tumor volumes [48]. Further exploration of the correlations between SVZ contact on MRI, FET PET scans, tumor volume and prognosis of glioblastoma patients may further elucidate the biomechanical backgrounds of SVZ contact in glioblastoma.

Deep-seated tumors present an increased surgical risk [49], and are more likely to be biopsied rather than resected as compared to superficial glioblastomas. In our cohort, periventricular tumors were indeed more often biopsied than superficial ones. Tumor resection is known to improve survival in glioblastomas, as compared to biopsies [50, 51]. Aside from their potential lethal consequences, complications could also delay or prevent optimal post-operative adjuvant cytotoxic treatment. In our cohort as well, patients with an SVZ contacting tumor experienced more serious (CTCAE grade 3–5) complications. At our center, the main criterion for glioblastoma patients to come in consideration for adjuvant combined therapy is having a KPS score > 70. Our patients with a preoperative KPS≥70 and an SVZ-contacting tumor were however ultimately significantly less often allocated to chemoradiation (as opposed to less optimal monotherapies) after surgery than patients with a glioblastoma without SVZ contact (χ^2^-test, P<0.0001), as a result of a decrease of KPS.

Given that SVZ-contact remained an independent prognostic factor in our multivariable survival analyses taking the type of surgery, post-operative complications and adjuvant treatment into account, other factors must contribute to the survival disadvantage of ventricular contact.

It has been suggested that SVZ-contacting glioblastomas present different biological characteristics than tumors without SVZ contact. Based on mRNA arrays, differential expression level of VEGF, HGF, CHI3L1, RAP2A, HES4, DLL3, PIR and HJURP, of several oncological transcriptomic (Notch, stem cell, hypoxia, angiogenesis and invasion) and inflammatory signatures have for instance variably been reported [16, 22, 25, 26]. An increased expression of mesenchymal markers (VEGF, HGF) was also observed by targeted q-RT PCR in SVZ-contacting tumors and differential expression of NOTCH1, CD133 and CHI3L1 was found between distinct periventricular regions[25]. A proteomic analysis of SVZ contacting tumors has also suggested a differential expression of vimentin, RBP1 and Lupus La, as well as an association with carbohydrate metabolism, blood coagulation, protease inhibitors and ECM pathways [24]. Our gene and miRNA expression analyses performed on proprietary samples and TCGA tumors, i.e. the largest cohorts analyzed to this end so far, could not replicate any of these results. In fact, even the findings of miRNA (n = 67) and gene expression (n = 71) analyses on our proprietary samples could not be reproduced in the larger TCGA/TCIA cohorts. This underscores the exploratory value of gene expression analyses and GSEA when performed on small or intermediate-size cohorts and the importance to validate them with other techniques or on larger samples prior to drawing any conclusions.

Steed et al. also observed that proneural and neural tumors from the TCIA grew frequently closer (as measured by the distance to those structures) to the SVZ than mesenchymal and classical ones [52]. Using the same dataset however and our clinician-friendly definition of SVZ contact based on the enhancing tumor core rather than a complex image analysis algorithm, we did not find any association between molecular subtype and SVZ contact in this same TCGA/TCIA and our cohorts. Our results are in line with a recent report of similar analyses to explore this hypothesis with the TCGA/TCIA dataset [46].

A limitation of gene expression analyses is the dependence on the presence of sufficient tumor material, favoring the recruitment of samples from debulking surgeries rather than from needle biopsies. Both our proprietary fresh-frozen samples and the TCGA samples indeed consist of surgical debulking tumor samples, without any needle biopsy samples. Biopsies were however significantly overrepresented in SVZ-contacting glioblastomas (41.5% versus 22.9%, Table 1, P<0.0005) in our consecutive patient cohort, and expression analyses may thus underrepresent the most aggressive/inoperable tumors. To minimize this bias, we also analyzed protein expression/activation patterns on TMAs that included the tumor tissues obtained from both biopsies and open surgeries of a subset of 220 consecutive patients. These analyses did not provide evidence for any association between SVZ contact and the expression of neural stem cell markers (CD133[35] and GFAP-δ[36]). This finding is congruent with a previous report [16], but contradicts another one based on microarray data [26]. Our gene set enrichment analyses suggested that SVZ contact could associate with increased NF-κB activity in glioma. This was however not confirmed at a protein level, as nuclear phospho-p65 and c-Rel protein expression did not differ between groups. In contrast, our proteomic analyses showed higher activation of C/EBP-β and STAT3 signaling in tumors contacting the SVZ. These transcription factors are hallmarks of (epithelial-)mesenchymal transformation in glioma, which is associated with poor prognosis in glioblastoma [34, 53, 54], and might prevail in SVZ-contacting glioblastomas compared to tumors without SVZ contact. Further research is needed to establish whether these observations represent intrinsic biological properties of tumors contacting the SVZ or are regional effects mediated by the SVZ microenvironment.

Due to the retrospective nature of our study, some established prognostic factors such as MGMT methylation status, Mini Mental State Exam (MMSE) score and use of corticosteroids [36] could not be included in our Cox model, as these data were too often unavailable. In addition, the patients in our study were diagnosed based on the WHO 2007 Classification of Tumours of the Central Nervous System [55] and IDH1 mutational status was only available for a subset of the patients, as it was not yet evaluated in routine clinical care. However, in a subset analysis, an IDH1 mutation was found in only 21 (6.1%) of 343 glioblastoma patients, and did not correlate to tumor contact with the SVZ. Based on these observations and the other baseline characteristics, we expect that the study cohort and study results are representative for patients with glioblastoma, IDH1 wildtype, according to the new classification system [56].

Despite these shortcomings, our cumulative results show that contact with the SVZ correlates with increased expression of markers of epithelial-mesenchymal transformation of glioblastomas, and is a significant adverse prognostic factor in these tumors, independent of age, performance status, tumor volume, type of surgery, postoperative complications and adjuvant treatment. We found no correlations between SVZ contact and molecular subtype, distinct gene expression patterns, or markers of stem cellness.

## Supporting information

S1 Appendix Clinical dataset. (XLSX)

S2 Appendix Supplementary methods. (PDF)

S1 Fig Molecular classification. No significant difference in molecular subclass distribution was detected between SVZ contacting glioblastomas and tumors not contacting the SVZ in the UMCU cohort (Fisher’s exact test, P = 1.0) and the TCGA cohort (χ2-test, P = 0.11).
(PDF)

S2 Fig RNA expression analysis UMCU cohort. Gene expression patterns of the 1000 RNA microarray probes with the highest standard deviation in the UMCU cohort. Gene expression patterns do not cluster to SVZ status or batch. No significantly differentially expressed genes were observed after correction for multiple testing (FDR < 0.05).
(TIF)

S3 Fig RNA expression analysis TCGA cohort. Gene expression patterns of the 1000 RNA microarray probes with the highest standard deviation in the TCGA cohort. Gene expression patterns do not cluster to SVZ status or batch. No significantly differentially expressed genes were observed after correction for multiple testing (FDR < 0.05).
(TIF)

S4 Fig MiRNA expression analysis UMCU cohort. MiRNA expression patterns of the 100 probes with the highest standard deviation in the UMCU cohort. 67 samples from our institute were included in this analysis. miRNA expression patterns did not cluster to SVZ status. No differentially expressed miRNAs were observed after correction for multiple testing (FDR < 0.05).
(TIF)

S5 Fig MiRNA expression analysis TCGA cohort. MiRNA expression patterns of the 100 probes with the highest standard deviation in the TCGA cohort. miRNA expression patterns did not cluster to SVZ status. No differentially expressed miRNAs were observed after correction for multiple testing (FDR < 0.05).
(TIF)

S1 Table Gene set enrichment analysis. Gene set enrichment in glioblastomas without SVZ contact compared to glioblastomas with SVZ contact. RNA expression data from the UMCU cohort (upper panel) and TCGA dataset (lower panel) was used. Abbreviations: ES: enrichment score; FDR: false discovery rate.
(DOCX)
