# Supplementary material for: Adverse prognosis of glioblastoma contacting the subventricular zone: Biological correlates
Source: PLoS One. 2019 Oct 11;14(10):e0222717. doi: 10.1371/journal.pone.0222717 (PMC6788733; doi:10.1371/journal.pone.0222717)
Supplement: S2 Appendix — (PDF) [file pone.0222717.s002.pdf]

Adverse prognosis of glioblastoma contacting the subventricular zone: biological correlates – S2 Appendix

Supplementary methods

Assessment of SVZ contact on MRI

For the first series of MRI scans (n=86), direct contact of the contrast-enhancing part of the tumor with the lateral ventricles was assessed by a resident in radiology and an experienced neuroradiologist on preoperative gadolinium-enhanced T1-weighted MRI scans blinded to the clinical data. Based on the high interobserver agreement (κ=0.78), the remaining MRI scans were assessed by one observer.

Fresh-frozen tissue samples and mRNA expression analysis

76 fresh-frozen surgical samples of *de novo* glioblastomas were prospectively collected between 2010 and 2015. RNA was extracted with the Nucleospin® TriPrep (Macherey-Nagel, Düren, Germany) and the QIASymphony RNA (Qiagen, Venlo, The Netherlands) kits according to the manufacturers' instructions. Affymetrix HG U133 plus 2.0 arrays were prepared and scanned according to the manufacturer's protocol and as reported previously [1, 2]. Quality control and differential gene expression analyses were performed with R (v3.2.2). After quality control, 71 samples were evaluable for analysis. Batch correction was performed with the 'sva' package. Exploratory Gene Set Enrichment Analyses (GSEA) were performed after RMA-normalization [3] and batch correction, with the Partek® Genomics suite platform (Partek v6.6, St. Louis, MO, USA). Analyses were performed with the Broad Institute MySig libraries of curated gene sets C1 – C7 version 5.0 [4], 1000 permutations and

default additional parameters. A FDR threshold of 0.25 was applied as recommended [3].

Class prediction

Molecular subclassification (proneural, neural, classical, mesenchymal) was predicted by hierarchical clustering [5]. Microarray normalization, data filtering and analysis of inter-array homogeneity were performed as reported previously [1, 5, 6]. Affymetrix HG U133 plus 2.0 probesets were matched to 840 genes originally published for the classification of glioblastomas (http://tcga-data.nci.nih.gov/docs/publications/gbm_exp/). Relative gene expression values were calculated. Genes were then excluded for a median absolute deviation below 0.5 [5]. After filtering, 768 genes were used for the class prediction. The hierarchical clustering of samples was performed with cluster3 software [7] with the agglomerative average linkage for the structure and 1 minus the Pearson's correlation for the distance metric [5]. Classes were unequivocally assigned to 62/76 samples. Differential distributions were tested with Fisher's exact test.

miRNA isolation and expression analysis

Total RNA was isolated from 76 fresh-frozen surgical samples of GBM patients with the MiRNeasy Micro Kit (Qiagen, Venlo, The Netherlands). Expression profiling of 800 miRNA probes was performed with the nCounter® Human v2 miRNA Expression Assay (NanoString Technologies, Seattle, WA, USA) at The Ohio State University Nucleic Acid Core Facility. Procedure details are available in the supplementary methods. 250ng RNA was used per sample and conditions were set according to the manufacturer's instructions. RNA quality was insufficient for 4 samples and SVZ status

was not available for 5 patients, leaving 67 samples evaluable for analysis. Data were processed with the Partek® Genomics suite platform (Partek v6.6, St. Louis, MO, USA) by geometric mean normalization, average background subtraction and normalization to housekeeping genes.

Copy number analysis

Total DNA was extracted with sufficient quality from 67 fresh-frozen samples from our proprietary cohort of glioblastomas and processed on SNP 6.0 Affymetrix chips as recommended by the manufacturer. After discarding one outlier on the PCA analysis of the raw intensity data, copy number analysis was performed on 66 samples after batch correction using the circular binary segmentation algorithm of the Partek Suite (35 with SVZ contact and 31 without SVZ contact, Partek v6.6, St. Louis, MO, USA). Copy number data were compared between the two groups using Chi-square tests and FDR correction for multiple testing.

Analysis of TCGA RNA and miRNA expression data

Preoperative gadolinium-enhanced T1-weighted MRI scans from all available TCGA-glioblastoma patients (n=262) were downloaded from The Cancer Imaging Archive (TCIA; accessed September 2015), and the SVZ status of these patients was determined as described above, blinded to the clinical data. Clinical and MRI data from 222 glioblastoma patients was available for Cox regression analysis.

Molecular classification data was downloaded from the UCSC cancer genomics browser (September 2015) and was analyzed for 228 patients. Clinical data and Level 1 Affymetrix U133A mRNA microarray data were obtained from the TCGA data portal ([8], September 2015). Differences in molecular subtypes between the tumor groups

were analyzed with a χ^2-test. After quality control, microarray data from 223 patients were analyzed with R, as described above. Correction for batch effects and GSEA were performed as described above. Heatmaps were created with the 'heatmap3' package (R).

Level 3 miRNA expression data from 236 glioblastoma patients was downloaded from the TCGA data portal (December 2015). MiRNA expression levels in glioblastomas with and without SVZ contact were analyzed with the 'limma' and 'heatmap3' package (R).

Tissue microarrays, immunohistochemistry and scoring

Archival FFPE tumor tissues were retrospectively collected for a consecutive cohort of 229 glioblastoma patients treated in the UMCU between 2005 and 2008. Tissue was available for inclusion in tissue microarrays (TMAs) for 220/229 patients and was processed as reported previously [1, 9]. SVZ status was unavailable for 14 patients.

Histology of each specimen was reviewed under supervision of a senior clinical neuropathologist and marked on H&E stained sections. Immunohistochemistry was performed, as described previously [1], with antibodies against c-Rel (Mouse monoclonal, Santa Cruz Biotechnology, Dallas, TX, USA), NF-κB p65 (phospho S276) (Rabbit polyclonal, Abcam, Cambridge, UK), STAT3 (phospho Y705) (Rabbit monoclonal, Cell Signaling, Leiden, The Netherlands), anti-C/EBPβ (Mouse monoclonal, Abcam, Cambridge, UK), anti-CD133 (Rabbit polyclonal, Rockland, Limerick, PA, USA) and anti-GFAPδ (Rabbit polyclonal, Netherlands Institute for Life Sciences, Amsterdam, The Netherlands [10, 11]). Antigen retrieval was achieved by incubation in citrate buffer (NF-κB p65, c-Rel, STAT3, C/EBPβ and GFAP-δ) or EDTA buffer (CD133) during 12 minutes at 126°C (NF-κB p65, c-Rel, STAT3,

C/EBPβ and CD133) or in a steamer for 30 minutes (GFAP-δ). Following incubation with the secondary antibody, the signal was developed with 3,3'-diaminobenzidine (DAB). Slides stained for GFAP-δ were incubated with anti–Rabbit Alexa 594 (Jackson Immunoresearch, Ely, UK). Nuclear counterstaining was performed with hematoxylin or Hoechst (GFAP-δ staining). In order to block auto-fluorescence, slides were incubated for 5 minutes in Sudan Black and thereafter rinsed with 70% ethanol.

Protein expression evaluation was blinded to the clinical data and supervised by a senior neuropathologist. The percentage of nuclear and/or cytoplasmatic staining was scored as: 0, negative; 1, 1-25% positive cells; 2, 26-50% positive cells; 3, 51-75% positive cells and 4, 76-100% positive cells.

The GFAP-δ stained tissue was analyzed with fluorescent imaging. ImageJ was used to calculate the percentage of positive GFAP-δ staining per sample. Auto-fluorescent and necrotic areas were excluded from the analysis. A mean staining score was computed for each patient. Staining scores were analyzed with Mann Whitney U tests.

Survival analyses

Statistical analyses were performed with use of SPSS 25.0 (IBM, Armonk, NY, USA). Two-sided *P*-values < 0.05 were considered significant. Kaplan-Meier curves were analyzed with the log-rank test. Cox regression was used for the survival analyses. The proportional hazards (PH) assumption of the Cox model was checked with log-minus-log plots and time-dependent variables. Details on this procedure have been published in a previous study [9]. The PH assumption did not hold for the KPS and tumor volume variables, suggesting that the hazard function of these variables change over time. Therefore, the model was extended with time-dependent variables for KPS (KPS*time)

and tumor volume (tumor volume*time). The PH assumption did not hold for the SVZ variable for patients with a long follow-up (>1000 days). The analyses were therefore restricted to a maximal follow-up of 1000 days after surgery.

Univariable Cox regression was performed to assess the prognostic effect of tumor contact with the SVZ on glioblastoma patient survival. Next, multivariable Cox regression was performed including the following variables: SVZ status, age, KPS, KPS*time, tumor volume, tumor volume*time, surgery type, and post-surgical adjuvant treatment.

595 patients could be included in a multivariable complete case analysis. Multiple imputation was performed as a sensitivity analysis, allowing inclusion of all 647 patients.

Survival analyses with TCGA data were performed as described above. Multivariable Cox regression was performed including age and KPS variables in the model. Multiple imputation was performed for missing data, to include all 222 patients of which survival and MRI data was available.

Supplementary references

1. Berendsen S, Spliet WGM, Geurts M, Van Hecke W, Seute T, Snijders TJ, et al. Epilepsy Associates with Decreased HIF-1alpha/STAT5b Signaling in Glioblastoma. Cancers. 2019;11(1). Epub 2019/01/10. doi: 10.3390/cancers11010041. PubMed PMID: 30621209.

2. Turkheimer FE, Roncaroli F, Hennuy B, Herens C, Nguyen M, Martin D, et al. Chromosomal patterns of gene expression from microarray data: methodology, validation and clinical relevance in gliomas. BMC Bioinformatics. 2006;7:526. doi: 1471-2105-7-526.

3. Subramanian A, Tamayo P, Mootha VK, Mukherjee S, Ebert BL, Gillette MA, et al. Gene set enrichment analysis: a knowledge-based approach for interpreting genome-wide expression profiles. Proceedings of the National Academy of Sciences of the United States of America. 2005;102(43):15545-50. doi: 0506580102.

4. Reich M, Liefeld T, Gould J, Lerner J, Tamayo P, Mesirov JP. GenePattern 2.0. Nature Genetics. 2006;38(5):500-1. doi: ng0506-500.

5. Verhaak RG, Hoadley KA, Purdom E, Wang V, Qi Y, Wilkerson MD, et al. Integrated genomic analysis identifies clinically relevant subtypes of glioblastoma characterized by abnormalities in PDGFRA, IDH1, EGFR, and NF1. Cancer Cell. 2010;17(1):98-110. doi: 10.1016/j.ccr.2009.12.020.

6. Wislet-Gendebien S, Poulet C, Neirinckx V, Hennuy B, Swingland JT, Laudet E, et al. In vivo tumorigenesis was observed after injection of in vitro expanded neural crest stem cells isolated from adult bone marrow. PloS One. 2012;7(10):e46425. doi: 10.1371/journal.pone.0046425.

7. Eisen MB, Spellman PT, Brown PO, Botstein D. Cluster analysis and display of genome-wide expression patterns. Proceedings of the National Academy of Sciences of the United States of America. 1998;95(25):14863-8.

8. Brennan CW, Verhaak RG, McKenna A, Campos B, Noushmehr H, Salama SR, et al. The somatic genomic landscape of glioblastoma. Cell. 2013;155(2):462-77. doi: 10.1016/j.cell.2013.09.034.

9. Berendsen S, Varkila M, Kroonen J, Seute T, Snijders TJ, Kauw F, et al. Prognostic relevance of epilepsy at presentation in glioblastoma patients. Neuro-Oncology. 2015. doi: nov238.

10. Roelofs RF, Fischer DF, Houtman SH, Sluijs JA, Van Haren W, Van Leeuwen FW, et al. Adult human subventricular, subgranular, and subpial zones contain astrocytes with a specialized intermediate filament cytoskeleton. Glia. 2005;52(4):289-300. doi: 10.1002/glia.20243.

11. van Strien ME, Sluijs JA, Reynolds BA, Steindler DA, Aronica E, Hol EM. Isolation of neural progenitor cells from the human adult subventricular zone based on expression of the cell surface marker CD271. Stem Cells Translational Medicine. 2014;3(4):470-80. Epub 2014/03/08. doi: 10.5966/sctm.2013-0038. PubMed PMID: 24604282; PubMed Central PMCID: PMCPMC3973708.
