# Supplementary figures and images for: Adverse prognosis of glioblastoma contacting the subventricular zone: Biological correlates
Source: PLoS One. 2019 Oct 11;14(10):e0222717. doi: 10.1371/journal.pone.0222717 (PMC6788733; doi:10.1371/journal.pone.0222717)

UMCU cohort

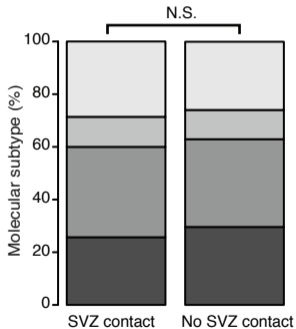

TCGA cohort

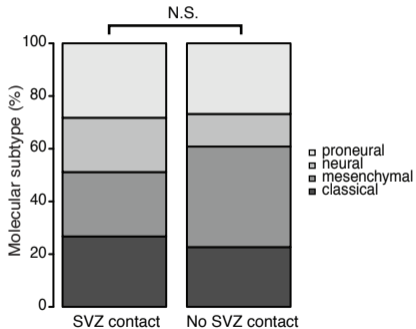

Supplement: S1 Fig — No significant difference in molecular subclass distribution was detected between SVZ contacting glioblastomas and tumors not contacting the SVZ in the UMCU cohort (Fisher’s exact test, P = 1.0) and the TCGA cohort (χ2-test, P = 0.11). (PDF) [file pone.0222717.s003.pdf]

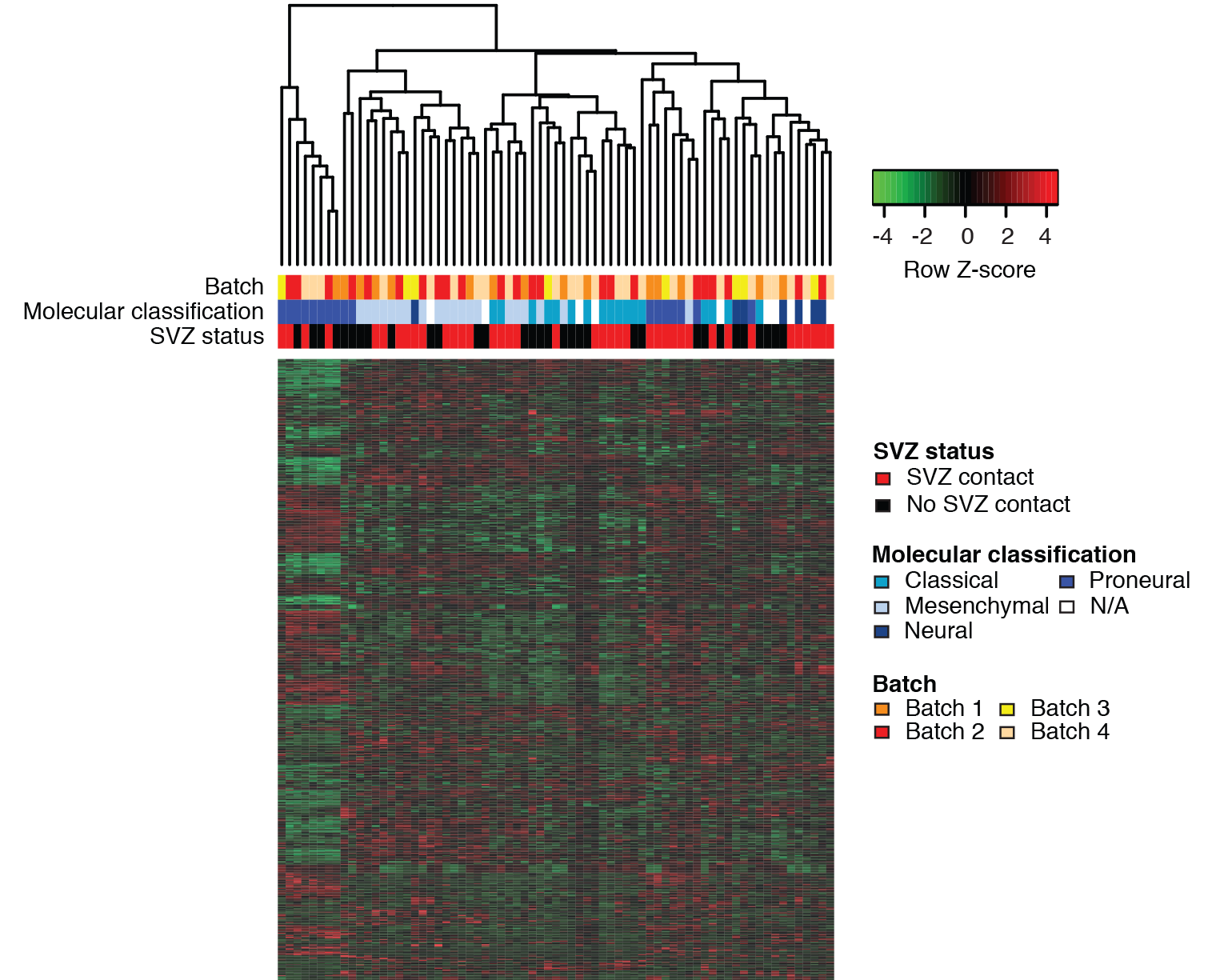

Supplement: S2 Fig — Gene expression patterns of the 1000 RNA microarray probes with the highest standard deviation in the UMCU cohort. Gene expression patterns do not cluster to SVZ status or batch. No significantly differentially expressed genes were observed after correction for multiple testing (FDR < 0.05). (TIF) [file pone.0222717.s004.tif]

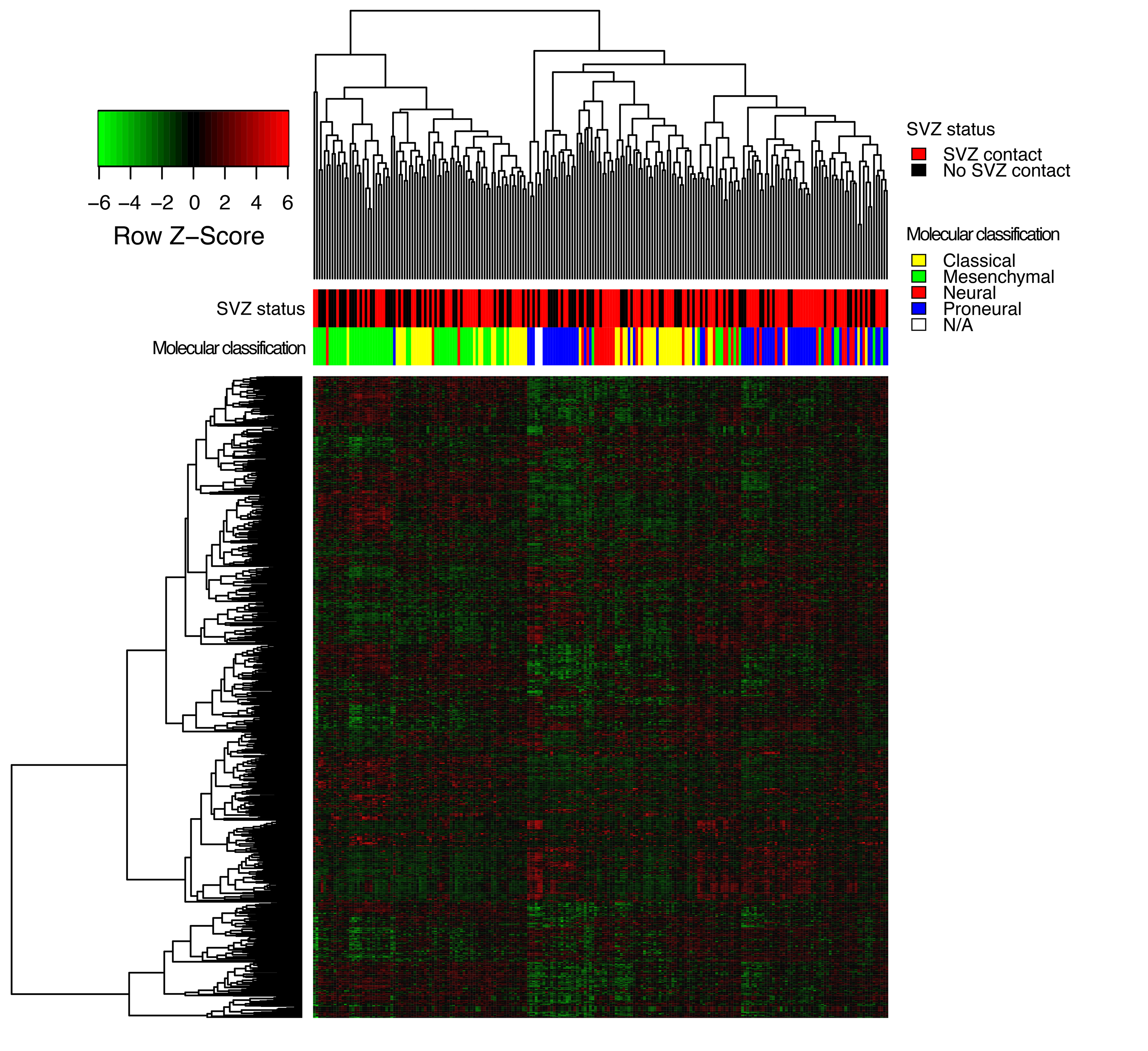

Supplement: S3 Fig — Gene expression patterns of the 1000 RNA microarray probes with the highest standard deviation in the TCGA cohort. Gene expression patterns do not cluster to SVZ status or batch. No significantly differentially expressed genes were observed after correction for multiple testing (FDR < 0.05). (TIF) [file pone.0222717.s005.tif]

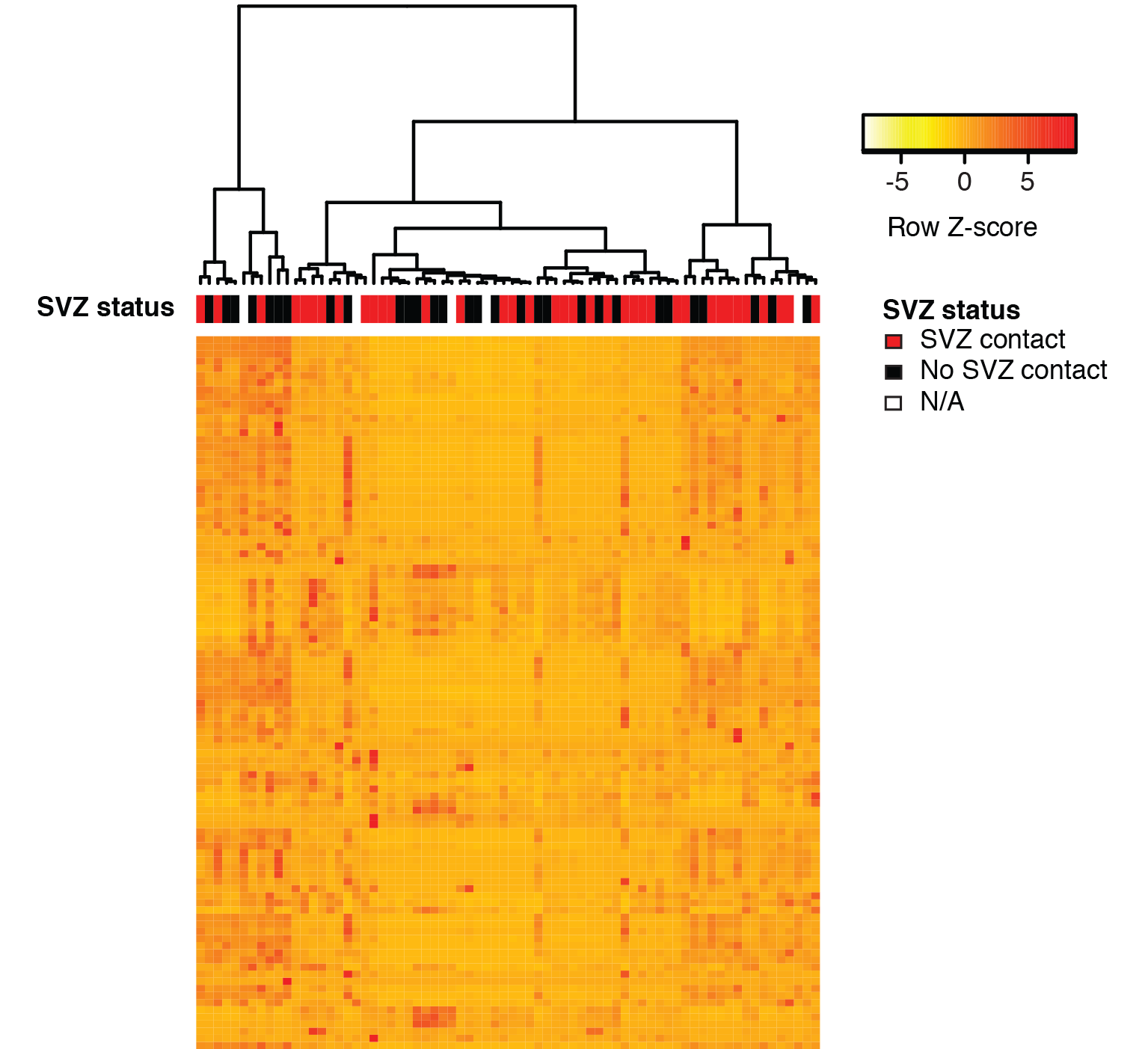

Supplement: S4 Fig — MiRNA expression patterns of the 100 probes with the highest standard deviation in the UMCU cohort. 67 samples from our institute were included in this analysis. miRNA expression patterns did not cluster to SVZ status. No differentially expressed miRNAs were observed after correction for multiple testing (FDR < 0.05). (TIF) [file pone.0222717.s006.tif]

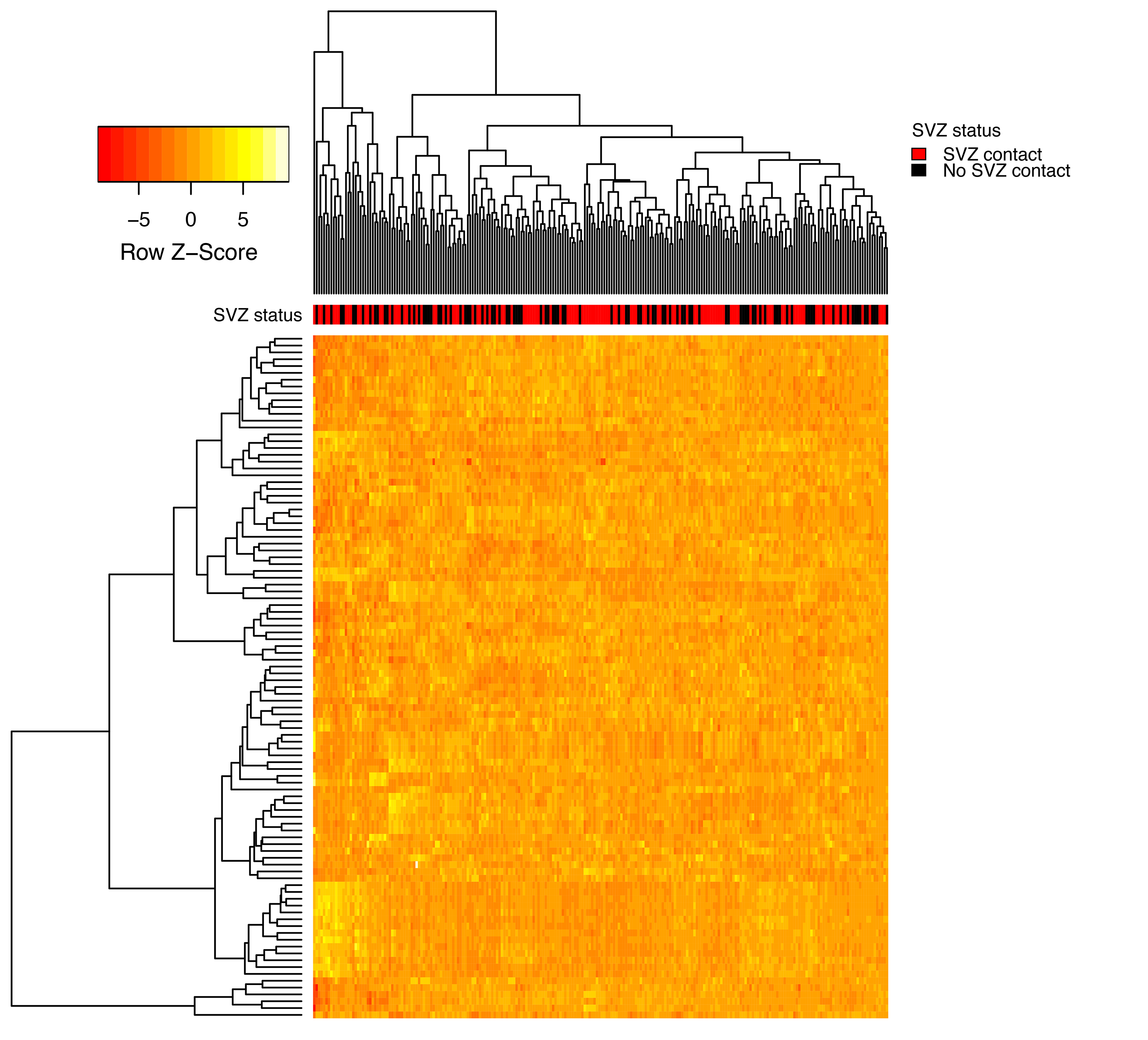

Supplement: S5 Fig — MiRNA expression patterns of the 100 probes with the highest standard deviation in the TCGA cohort. miRNA expression patterns did not cluster to SVZ status. No differentially expressed miRNAs were observed after correction for multiple testing (FDR < 0.05). (TIF) [file pone.0222717.s007.tif]
