# Supplementary material for: Adverse prognosis of glioblastoma contacting the subventricular zone: Biological correlates
Source: PLoS One. 2019 Oct 11;14(10):e0222717. doi: 10.1371/journal.pone.0222717 (PMC6788733; doi:10.1371/journal.pone.0222717)
Supplement: S1 Table — Gene set enrichment in glioblastomas without SVZ contact compared to glioblastomas with SVZ contact. RNA expression data from the UMCU cohort (upper panel) and TCGA dataset (lower panel) was used. Abbreviations: ES: enrichment score; FDR: false discovery rate. (DOCX) [file pone.0222717.s008.docx]

| **UMCU cohort** |  |  |  |  |
| --- | --- | --- | --- | --- |
| **MSigDB collection** | **Gene set** | **ES** | ***P*-value** | **FDR** |
| C1 – positional gene sets | chr9q34 | 0.57 | <0.001 | 0.038 |
| C3 – motif gene sets  *Transcription factor targets* | GCGNNANTTCC_UNKNOWN | 0.37 | 0.002 | 0.229 |
|  | GTCNYYATGR_UNKNOWN | 0.35 | <0.001 | 0.239 |
|  |  |  |  |  |
| **TCGA cohort** |  |  |  |  |
| **MSigDB collection** | **Gene set** | **ES** | ***P*-value** | **FDR** |
| C1 – positional gene sets | chr3p24 | -0.69 | <0.001 | 0.070 |
|  | chr3q22 | -0.68 | 0.006 | 0.092 |
|  | chr3q29 | -0.65 | 0.006 | 0.113 |
|  | chr3q23 | -0.73 | 0.004 | 0.118 |
|  | chr3q27 | -0.63 | 0.006 | 0.127 |
|  | chr3p25 | -0.54 | 0.006 | 0.134 |
|  | chr3q26 | -0.53 | 0.004 | 0.182 |
|  | chr3q28 | -0.66 | 0.016 | 0.184 |
|  | chr19p12 | -0.65 | 0.018 | 0.217 |
| C3 – motif gene sets  *Transcription factor targets* | V$PEA3_Q6 | 0.46 | 0.004 | 0.141 |
| C6 – oncogenic signatures | BMI1_DN.V1_UP | 0.48 | 0.004 | 0.185 |
